# Medical Cyclopedia

**Published:** 1835

**Authors:** 


### Art. VIII. — Medical Cyclopedia.

*The American Cyclopedia of Practical Medicine and Surgery, Part IX.* Carey, Lee and Blanchard, Dec. 1835.

We have lately received the 9th part or number of this comprehensive and learned work. It is in a great degree composed of articles from the pen of the erudite and laborious Professor Geddings, of Baltimore, whose cultivation of morbid anatomy bids fair to render him an ornament to the American Profession. His contributions in this number are chiefly on the special anatomy and pathology of the arteries. As usual his articles indicate a variety of research, and may be referred to as authority. As a specimen, we shall select the diagnosis and treatment of inflammation of the arteries.

"*Symptoms and Diagnosis of Arteries.*—The symptoms of acute inflammation of the arteries are so analogous to those common to many other affections, that it is not easy to discover any traits sufficiently constant and characteristic, to distinguish it from diseases of other portions of the organization. The difficulty is greatly augmented by the deep seat of some of the principal vessels, by the complex relations of all of them, and by their peculiar liability to have their functions thrown into disarray, by almost every disturbance affecting other tissues and organs. We shall first enumerate the principal symptoms that accompany the disease in its different stages and modifications, and afterwards consider how far any of them can be relied on in forming a diagnosis.

Acute inflammation occupying a small extent merely of a single artery, is often unattended with any very striking disturbance of either a local or general character. Thus, after the application of a ligature, which always occasions more or less inflammation of the vessel, there is often no very palpable alteration of the part: there is but little increase of sensibility, heat, or tumefaction, and the whole process of obliteration may be accomplished, without the supervention of any febrile or other constitutional disturbance. It is far different, however, when the inflammation is more extensively diffused, and when it seizes with considerable intensity upon a large extent of the arterial system. Under such circumstances, it is attended with violent fever of the inflammatory kind, the symptoms of which vary much in the progress of the disease. The modifications thus accruing, are partly attributable to the influence exerted upon the vital powers, and in part to changes taking place in the structures of the vessels themselves, and the blood they circulate. The first symptoms manifesting a general disturbance of the system, are commonly slight chills, alternating with flushes of heat, which are speedily followed by intense fever, attended with violent pulsations of the heart and arteries, a red suffusion with capillary injection of the skin, burning heat, unquenchable thirst, extreme restlessness, jactitation and general distress, laborious and hurried respiration, and sometimes a dry harassing cough. When the inflammation is seated in the aorta, there is, according to Joseph Frank, a sense of heat beneath the sternum, or in the vicinity of its upper extremity; and Spangenberg and Kreysig remark, that the patient sometimes complains of a sensation like that of hot iron drawn along the course of the aorta, descending as low as the crural arteries. When the arteries of the extremities are affected, there is pain, sometimes simulating rheumatism, sometimes of a burning kind, in the course of the inflamed vessel, which either fixes itself with considerable intensity upon a particular part, or is unequally diffused. The affected point is tender to the touch, and if the vessel be superficial, there is generally increased volume and hardness of its walls, perceptible through the integuments. There is likewise increased heat, and sometimes more or less tumefaction, with a distressing sense of throbbing. At first the pulsation is preternaturally violent along the whole extent of the inflamed artery, but in proportion as its tunics, and the blood within, become altered, the inordinate throbbing and expansion of the vessel is confined to the portion above the principal seat of the inflammation, while lower down the pulsation becomes feeble, and when obliteration takes place, finally ceases altogether. Should the carotid artery be affected, there will be pain and tenderness along the side of the neck, attended with violent pulsation in that region and in all the arteries of the head,—distressing cephalalgia, vertigo, flashes of light before the eyes or obscuration of vision, ringing or roaring in the ears, and in some cases violent delirium, convulsions, or even apoplexy. In one of Frank's cases, the patient complained of every thing wearing a green color; and in most instances, whatever artery is affected, the corresponding veins and capillaries are preternaturally turgid in the first stage of the disease, owing to the blood being propelled into them with increased impetuosity.

From the onset of the disease, the heart beats violently, and is affected with frequent palpitations, especially if the inflammation implicates its lining membrane. There are forcible pulsations or throbbings of the whole arterial system, and during the whole of the first stage, the pulse is full, strong, tumultuous, bounding, and sometimes exceedingly frequent. J. P. Frank remarks, that it is in some instances as tense and as hard as a metal wire; and in one of the cases observed by him, its beats fluctuated from 185 to 200 in a minute. It also frequently intermits, or presents a kind of double stroke, and in some instances, if blood be drawn, both the hardness and frequency are increased. The respiration is hurried, embarrassed, and occasionally so difficult as to amount to distressing orthopnœa. There is also a troublesome dry cough, and sometimes hemoptysis; and if the disease be seated in the aorta, any sudden movement of the body occasions an increase of pain, and augments the general suffering. The tongue is red on the edges, its papillæ prominent, and its base and middle coated with a thick fur. The thirst is intense, and unquenchable; the stomach sometimes irritable; the bowels constipated, and the urine high colored, scanty, and voided with a sense of scalding. It is occasionally dark colored, turbid, or even bloody. The face and eyes are suffused and turgid; blood occasionally flows from the nose; the injection of the capillaries occasions a preternatural turgescence of the skin and all the superficial vessels, and the surface sometimes assumes a deep red, purplish, or mottled appearance.

"As the disease advances, the vital powers are gradually exhausted under its ravages; the coats of the inflamed arteries undergo important alterations; the blood sometimes becomes coagulated in the affected vessels; and with a general aggravation of most of the above symptoms, a new train of phenomena is frequently superadded. The general anxiety, restlessness, and distress, now increase. The pulse augments in frequency; becomes small, wiry, weak, irregular, and acquires a peculiar sharp thrill. The dyspnœa is more distressing. The heart is affected with frequent palpitations, and the slightest motion or exertion, or an erect posture, occasions vertigo or a disposition to syncope. The tongue is dry, scabrous, and covered with a brown sordes; the thirst is intense; the skin is covered with clammy sweats; the countenance assumes a sunken, decomposed or cadaverous expression, or it is lurid and œdematous; the bodily powers decline, and the patient is affected with delirium, especially at night, subsultus, tendinum, or frequent convulsive movements of different parts of the body. The bowels, which are at first constipated, now assume an opposite condition, and the dejections are frequent, dark colored, and highly offensive. The urine is also dark, turbid, and fœtid, and the whole of the secretions and excretions are perverted. The skin, which was at first simply injected, now assumes a deeper purplish hue, and if the inflammation occupy one of the principal arteries of the extremities, the condition of the affected parts undergoes important changes. The alterations of the textures of the vessel, and the formation of fibrinous concretions by the coagulation of the blood within, gradually interrupt the transit of that fluid, and the portion of the member situated below the seat of the disease, has its nutritive functions impaired in a corresponding degree. The preternatural pulsation, which at first was manifest along the whole course of the artery, is now only perceptible above the seat of the disease, while below it is feeble and indistinct. This arises from the gradual obliteration of the vessel, and is attended with a sense of coldness and numbness of the part, resulting from the privation of its nutritive fluid. The obstacle to the course of the blood thus created, occasions a distention of the artery above, with preternatural pulsation; and Naumann remarks, that in one case, it imparted the sensation, to the finger, of a distended membranous sac, having a fluid issuing from its surface by numerous pores. (*Handbuch der Med. Klinik.* II. 606.) Should the obliteration be complete, all pulsation below ceases; the limb becomes œdematous; the blood gradually accumulates in the radicles of the veins, giving rise to a dark ecchymosed appearance, arranged either in isolated points, or more diffused; the part becomes cold and numb; small purplish phlyctenæ make their appearance, and the part finally falls into gangrene, unless the collateral anostomoses should so far escape the disease, as to admit of their furnishing a sufficient supply of the vital fluid to obviate these consequences. With the development of these phenomena, the powers of life become more prostrated; the pulse more frequent, small, sharp, and irregular; the clammy sweats more profuse; the delirium gives place to a profound coma; the evacuations come away involuntarily; hiccup supervenes; the extremities grow cold, and the patient either sinks exhausted, or death is ushered in by convulsions.

"When the disease takes a more favorable course, it is represented by Puchelt, that it sometimes pursues a regular progress until the seventh day, exacerbating twice in the twenty-four hours, and then forms a crisis, either by a free perspiration, hemorrhage, or the deposit of a copious sediment in the urine. (*System der Medicin.* Band II. II. Th. 63.)"

" *Treatment of acute inflammation of the Arteries.*—Much need not be said on the treatment of acute inflammation of the arteries. It must be varied according to the stage of the disease, its intensity, and the complications with which it is associated. In the active or sthenic forms of the disease, especially when the arterial system is extensively involved, the free abstraction of blood presents itself as the chief remedy, and it must be pushed to an extent commensurate with the exigencies of the case, and the powers of the system to bear it. Blood must be freely abstracted from the arm, and the operation repeated as often as the necessity may recur provided circumstances should not arise to render such copious depletion improper. Local depletion will also be found serviceable, especially when the affected vessel is superficial. Leeches should be freely applied along the course of the inflamed artery, and the bleeding encouraged by fomentations with warm water. Should one application not be sufficient, they must be repeated at proper intervals, until a sufficient effect is produced. It was remarked by Frank and Spangenberg, that the abstraction of blood rendered the pulse harder and more tense. This has been regarded by some practitioners as a sufficient reason for not resorting to the practice. But when the indications of active inflammation are unequivocal, bleeding is indispensable, and if the disease should be associated with a neuropathic disposition of the vessels, disposing them after venesection to take on this peculiar kind of reaction, when sufficient blood has been detracted, it may be useful, with the view to prevent the bad effects of this nervous erethism, to prescribe an opiate, either alone, or what will be preferable, combined with antimony and calomel, as recommended by Copland. But in resorting to the abstraction of blood, it must be borne in mind, that there are not unfrequently circumstances in the course of this disease, which will render it unsafe to push general bleeding to a great extent. There is frequently much proneness to syncope even after a slight bleeding, and the inflammation of the arteries is besides only developed in many instances, during the last stage of inflammatory and ataxic fevers, small-pox, scarlatina, puerperal fever, etc., when the powers of life are so prostrated, that the withdrawal of even a small quantity of the vital fluid might be productive of fatal consequences. Still, when there is great tenderness in the course of an artery, especially if the artery be superficial, it may be necessary in some instances, even under these circumstances, to apply a few leeches along the course of the vessel—acting upon the same principles that guide us in the treatment of other local inflammations, attended with impairment of the vital powers.

"Simultaneously with the abstraction of blood, the patient should be put upon the use of mild saline cathartics, either alone or alternated with calomel;—nitrate of potash, antimonials, mild diluent, subacid drinks, cathartic enemata, and, when preternatural throbbings of the arteries and palpitations of the heart follow the abstraction of blood, large doses of opium, black drop, or hyoscyamus, may be given in combination with calomel and antimony, to which when the powers of life are considerably prostrated, camphor may be added. Mercury, pushed to the extent of producing slight soreness of the mouth, has been recommended by Hope, Berndt, and Copland. But from the tendency of this article to occasion preternatural excitement of the heart and arteries, when administered so as to produce its constitutional effects, we should be reluctant to employ it in that manner, except in those cases in which the arteritis is induced by syphilis. After sufficient depletion, Hildenbrand recommends the acetate of lead and digitalis, and from the power possessed by both of these agents in moderating the activity of the circulation, we doubt not they may be sometimes useful when employed with proper discrimination. Neither of them should be relied on to the exclusion of more important means, nor will they be proper in the very advanced stage of the disease, when typhoid symptoms have made their appearance. Colchicum has also been suggested by Copland; and when the intensity of the inflammation is considerably subdued, leaving a preternatural throbbing of the arteries, which is dependent on a state of morbid nervous erethism of those vessels, prussic acid might perhaps be employed with advantage.

"When the disease occupies a superficial artery, as for example, in the extremities, the neck, etc., after sufficient blood has been detracted by leeches, the part may be constantly covered with refrigerant and anodyne lotions, or if these should disagree, tepid ablutions may be employed with advantage. In addition to these applications, Berndt recommends, after the inflammation has lost its active character, the employment of warm moderately exciting aromatic fomentations, lotions of muriate of ammonia dissolved in vinegar and water, and frictions with mercurial ointment. (Rust's *Handb. der Chirurgie.* II. 298.) Under the same circumstances, a blister applied over the part, so efficacious in some cases of phlebitis, might possibly produce a good effect. Revulsives will also be useful in the advanced stages of inflammation of the large arteries of the splanchnic cavities. (See *Aorta.*)

"The various complications of the disease with the several forms of fever,—with erysipelas, gangrene, etc., must be managed upon the general principles proper to be observed in the treatment of those diseases. As these complications are seldom observed, except when the vital powers have become considerably prostrated, blood-letting to any considerable extent will seldom be admissible, and the chief reliance must be placed upon mild antiphlogistics, and those means which, by exciting the several emunctories to the performance of their proper functions, will tend to separate from the mass of the circulating fluids, those materials which are generated in the blood, and tend to irritate the vessels. When obliteration of the arteries has taken place, giving rise to gangrene, the case must be managed according to the rules laid down in the article *Mortification.*

"It is scarcely necessary to subjoin, that during the whole treatment, perfect quietude of both body and mind should be rigorously enjoined. The patient should be strictly confined to a light farinaceous diet, with iced, or cool demulcent drinks; and even during convalescence it will be necessary to restrict him from all animal food for a considerable time. Every source of irritation, both physical and mental, must, in short, be carefully avoided, since the heart and arteries are preternaturally susceptible, and owing to their intimate sympathies with the other organs, they will be seriously influenced by an active impression made upon any part of the system.

"The *treatment of chronic inflammation* of the arteries may be dismissed in a few words. As the disturbance it occasions is seldom observed until the disease has advanced so far as to occasion extensive transformation of the arterial tisues, its existence is not known while it is in a condition to be cured. When these transformations of texture have taken place, it is beyond the reach of our remedies, and all that can be done is to obviate or remove the inconveniences they may occasion. They are chiefly such as arise from impaired circulation, and when they give rise to congestions of the left side of the heart, or of other organs, these must be removed by repeated small abstractions of blood, mild aperients, a proper attention to diet, and an avoidance of the ordinary sources of irritation. In some cases, where there is a loss of tonicity, unattended with serious changes of structure, chalybeates, and other tonics, with the shower-bath, may be useful."

We take the liberty of making two suggestions to the enterprising publishers of the work before us. First, the wood cuts with which Dr. Geddings' Special Anatomy of the Arteries, is (nominally) illustrated, may fairly be considered as a disgrace to the article. Secondly, In the original prospectus it was stated, that the work, it was expected, would be comprised in 40 numbers, making eight volumes, the cost of which would be twenty dollars. Under this expectation, no doubt, many country physicians became subscribers, when if they had supposed the Cyclopedia would ultimately extend in price to 50 or 60 dollars, they would have declined. Now, the 9th part, making almost a fourth part of the whole of the work proposed, does not carry us through the letter A. At this rate of advancement, more than a hundred numbers will be required to complete the Cyclopedia; the publication of which will not be finished under several years. This augmentation of the limits originally prescribed, seems to us an inadmissible liberty; and one, moreover, which is attended with no particular advantage to the profession. Such a work ought, properly, to be a condensed book of reference; from which a great number of things to be found in all the standard works, might, with advantage, be excluded. We make these remarks in a spirit of perfect good will towards the Cyclopedia, and under the conviction, that it would be rendered more extensively useful, if they should meet with some attention.

